# The Book World of Medicine and Science

**Published:** 1897-11-27

**Authors:** 


### THE BOOK WORLD OF MEDICINE AND SCIENCE.

Practical Domestic Hygiene. By Surgeon-Colonel J. Lane Notter, M.A., M.D., Fellow and Member of Council of the Sanitary Institute, Examiner in Public Health in the University of Cambridge and in the Victoria University, Manchester, Professor of Hygiene in the Army Medical School, Netley; and Surgeon-Major R. H. Firth, F.R.C.S., Assistant Professor of Hygiene, Army Medical School, Netley, Surgeon-Major Army Medical Staff. (Longmans, Green, and Co., London, New York, and Bombay. 1897. 312 pages, 81 illustrations. 2s. 6d.)

Although this book is only given to us as a most elementary work on hygiene, it is none the less a veritable compendium of useful knowledge. It is written for non-professional persons, and calls itself a manual for domestic use. It is divided into three parts, severally devoted to questions of elementary human anatomy and physiology, elementary hygiene, and domestic economy. The first part contains an indispensable minimum of physiological data, illustrated with a number of excellent and time-honoured diagrams. The second and chief part, in addition to all the usual subjects, contains chapters on diseases due to food, and parasitic and infective diseases—chapters containing convincing illustrations of the true importance of their subjects from a domestic standpoint. In the section on house construction a few more observations on the cause and cure of smoking chimneys would have been acceptable, while in the section on personal hygiene the common bad results of excessive tobacco smoking, or of smoking bad tobacco, might with advantage have been pointed out. The third part of the book discusses the care and management of the sick, and in addition gives a *résumé* of the work usually taught in ambulance classes. The authors give their reasons for all they say with such clearness and fulness that there is not an uninteresting page in the book, and if there are any so confident of their knowledge that they would not wish to read it for instruction, they are none the less advised to read it for pure pleasure.

The Dwelling House. By George Vivian Poore, M.D., F.R.C.P., Physician to University College Hospital, Fellow of the Sanitary Institute, &c. (Longmans, Green, and Co., London, New York, and Bombay. 1897. Index, 178 pages, 36 illustrations. Price 3s. 6d.)

This book is for the most part a reproduction of addresses delivered, or papers communicated to various scientific bodies or to the *Practitioner*. It contains an indictment of our modern methods of sanitation. It also deals with the causes of the increasing overcrowding of our large centres of population, and with the train of social and sanitary ills which follow on the heavy taxation of the dwelling house. The book certainly gives the oppressed householder very little hope of mending life in London in any way short of ending it; it merely aggravates the misery of his lot by holding up to him a mirror, in which his deplorable plight is only too evident. A graphic picture is presented of the generally disproportionate, over-plumbered, under-ventilated London house. The chapters which follow on the disposal of sewage in country districts by the dry and other simple methods, together with the sanitation of cow-sheds, and the use of human and other excreta for agricultural purposes, are full of practical interest. The subject of overcrowding is fully discussed in relation to building societies, building regulations, model bye-laws, and, above all, to the cost of dwellings, and the root of the matter is demonstrated to lie in the increasing total obligatory charges in connection with the renting of houses. The great blot on modern sanitary legislation is the principle on which "the sanitary well-doer is heavily taxed for the support of the jerry builder, and is called upon to pay for all the shortcomings of the negligent and filthy." "Encouragement ought to be given to the man who builds a house with ample curtilage." The whole subject is a rousing one, and the book, besides deeply stirring the reader's social and sanitary instincts, leaves him with a sense of having had salt rubbed into sundry raw places on his own much-burdened back.

Memory and its Cultivation. By F. W. Edridge Green, M.D., F.R.C.S. International Scientific Series. (London: Kegan Paul, Trench, Trübner, and Co., Limited. 1897. 310 pages.)

This is not a book for the psychologist, but then it does not claim to be. The author in his preface tells us that he has discovered facts which have enabled him "to learn a subject in about a fifth of the time that it previously took," and gives his method to the public as a time and labour-saving appliance. In his introductory chapter the author announces that he will "demonstrate that memory is a definite faculty, and has its seat in the basal ganglia of the brain separate from but associated with all the other faculties of the mind." This raises the reader's expectations, but the subsequent proofs advanced are not particularly convincing. It is assumed at the outset that the brain is the "seat" of the mind. We are not, however, so much concerned in criticising conclusions for which no data are offered, as in objecting on any hypothesis to the use of "brain" and "mind" as convertible terms. Yet this confusing misuse continually occurs. Thus, "the brain is made up of a number of ultimate faculties," "the portion of the brain devoted to the sentiment of love." The reader is compelled to the suspicion that the author's theories are tinged by those of the phrenologist, and this is confirmed by his use of the phrenologist's classification of the ultimate faculties of the mind, and is not dispelled by the unnecessary length at which he argues against the phrenologist's localisation of function. Yet the writer's own arguments in favour of the localisation of memory read not unlike the arguments of this school. The necessary "requirements" for this theory to work, are first given, and then "the parts of the brain in a position to fulfil these requirements" are said to be—but in no way, we think, demonstrated to be—the optic thalami and the corpora striata. No mention is made of the inhibitory mechanism by which, it is to be presumed, such impressions as are not of immediate use are "switched off." Yet is it not probable that the process of forgetting or ignoring may be as active a function of mind as any other? It seems as though any study of a particular function is incomplete without some consideration of this point. The author gives three "laws of remembrance" and twelve rules for the improvement of the memory, the essence of which latter seems to lie in the cultivation of habits of attention.

